# Diagnostic accuracy evaluation of individual or combinational fecal immunochemical test, M3 gene, KRAS mutation and tumor methylation burden in colorectal carcinoma

**DOI:** 10.3389/fimmu.2025.1627130

**Published:** 2025-07-23

**Authors:** Junyue Xu, Shuai Chen, Hongxia Niu, Yahong Zhao, Xiaohong Wu, Yi Li, Shana Zhang

**Affiliations:** ^1^ Department of Clinical Laboratory, Capital Medical University Electric Power Teaching Hospital, Beijing, China; ^2^ Department of Otolaryngology-Head and Neck Surgery, The First Affiliated Hospital of Xiamen University, School of Medicine, Xiamen University, Xiamen, China; ^3^ Xiamen Key Laboratory of Otolaryngology Head and Neck Surgery, Xiamen, China; ^4^ Department of Emergency Medicine, Capital Medical University Electric Power Teaching Hospital, Beijing, China; ^5^ Department of Traditional Chinese Medicine, Capital Medical University Electric Power Teaching Hospital, Beijing, China; ^6^ Botnar Research Centre, Nuffield Department of Orthopaedics, Rheumatology and Musculoskeletal Sciences, University of Oxford, Oxford, United Kingdom

**Keywords:** colorectal carcinoma, fecal immunochemical test, M3, KRAS mutation, tumor methylation burden

## Abstract

**Objective:**

This study is aimed to develop multivariate prediction method in colorectal cancer (CRC) diagnosis.

**Methodology:**

M3 gene expression was determined using Fecal DNA extraction kits and performed by qRT-PCR. Methylation-burden and KRAS-mutation were detected by using the corresponding kits. Receiver operating characteristic curve analysis and the area under the curve (AUC) was calculated to evaluate diagnostic performance using SPSS software.

**Results:**

197 of CRC samples were enrolled to screen the best predictive combination among fecal immunochemical test (FIT), M3 expression and KRAS-mutation in feces, and Methylation-burden in blood. Single factor analysis showed that M3 expression showed the best diagnosis performance and fecal immunochemical test (FIT) showed the lowest AUC. Combination of two makers universally enhanced diagnostic performance, of which Methylation-burden and M3 alliance displayed the highest AUC value. Interestingly, combination of M3, Methylation-burden and KRAS-mutation reached the best performance for all patients (AUC: 0.920), especially for early CRC patients (AUC: 0.931), which possessed the same predictive efficiency with the combination of four factors.

**Conclusion:**

Combined application of M3, Methylation-burden and KRAS-mutation might be the most reliable method for early CRC diagnosis.

## 1 Introduction

Colorectal cancer ranks among the most prevalent malignant neoplasms (1, 2). According to the 2020 global cancer statistics, there were approximately 19.3 million new cancer cases and 9.9 million cancer-related deaths worldwide, with colorectal cancer being the third most common malignancy and the second leading cause of cancer-related mortality (3). Early detection and diagnosis are crucial for reducing the mortality of colorectal cancer (4). Currently, colonoscopy remains the gold standard for diagnosis of CRC, but its invasiveness and the complexity of bowel preparation are often deterrents for patients (5). To address these limitations, many patients are now interested in a two-step screening approach: first, a non-invasive fecal test, followed by a colonoscopy if the test is positive. However, non-invasive tests still often produce false positives for advanced adenomas and colorectal cancer. Therefore, the better non-invasive methods for early colorectal cancer diagnosis are needed.

Several diagnostic strategies for CRC have been widely developed, such as fecal immunochemical test (FIT), *M3* gene from *Lachnoclostridium* and tumor methylation burden (6, 7). FIT has been widely used because it is not affected by the daily diet (8). Adjusting the Hb cut-off value of quantitative fecal immunochemical test (qFIT, measuring fecal hemoglobin levels) can help determine the need for colonoscopy and assess neoplasia risk. Although qFIT is commonly used for early screening, its low sensitivity is a major challenge in clinical practice (9). According to a recent study, CRC cells with genetic and epigenetic changes are shed into the stool and their DNA alterations can be detected, which may contribute to improve the detection sensitivity of FIT (10–12).

Besides, KRAS mutation mainly in Glycine 12 and 13 have been found in 30-40% CRC patients (13). Several researches have pointed that early development of CRC is closely associated with the methylation of promoter regions in CRC-related genes, such as NDRG Family Member 4 (NDRG4) (14). The detection of NDRG4 gene methylation in stool has also been proposed as a potential diagnostic biomarker for CRC screening (15). In fact, the detection rates for CRC and precancerous lesions were 31.86% and 33.80% respectively through colonoscopy following a positive multi-target stool FIT-DNA test, which is significantly elevated compared to 15.82% with colonoscopy alone. The combination of FIT and stool DNA testing has been widely applied in clinical early screening for CRC (16). Recently, metagenomic studies identified a novel fecal genetic marker, the M3 gene from *Lachnoclostridium*, which is significantly enriched in CRC and adenomas (17). The combination of the M3 gene with qFIT testing improves the diagnostic sensitivity for advanced adenomas (sensitivity: 56.8%; specificity: 79.6%) (17). However, whether the combination of the fecal M3 marker and multi-target stool FIT-DNA testing can achieve better sensitivity and specificity requires validation through large-scale clinical trials. Although a variety of prediction models have been designed to predict the prognosis and mortality of patients with CRC (18), a comprehensive prediction model trusted by most medical workers with several clinical indicators such as FIT, M3 and methylation to effectively predict the disease development of CRC patients is still needed at the present.

This study aims to evaluate various contemporary clinical diagnostic markers for colorectal cancer and employ multifactorial analysis to elucidate their significance in CRC diagnosis. By leveraging multiple models, the objective is to develop a more reliable diagnostic strategy.

## 2 Materials and methods

### 2.1 Patients, sampling and measurements

**Figure 1:**
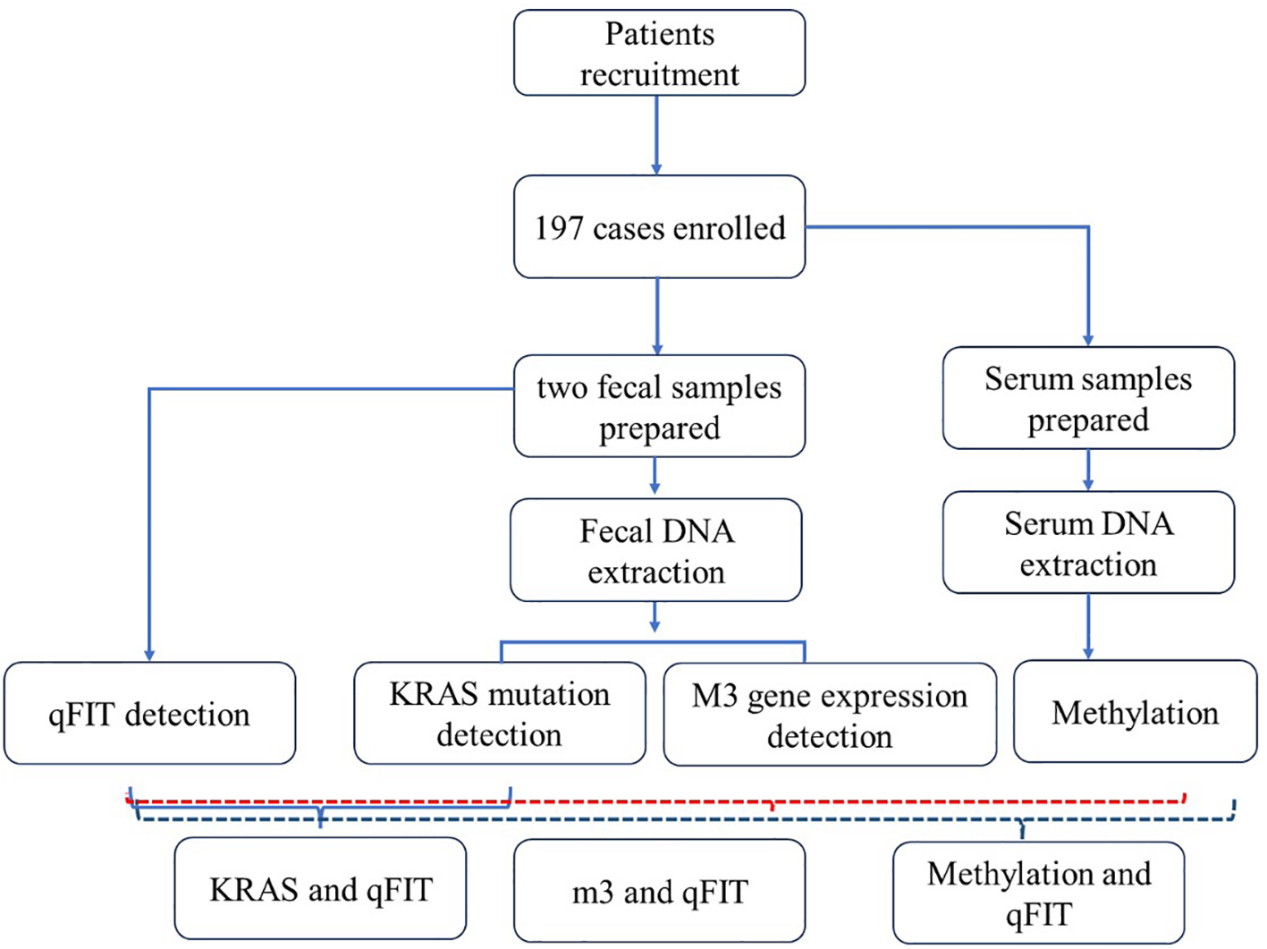
Workflow of this study.

This project collected 197 patients from Beijing Electric Power Hospital, Shanghai Electric Power Hospital, and Shandong Electric Power Hospital from the start of the project until six months before the project’s conclusion (30 August 2022 to 31 July 2024). The sample size was determined by G-power analysis. All experiments were approved by medical ethics committee of Capital Medical University Electric Power Teaching Hospital (2022083010101). All enrolled participants must undergo a colonoscopy within one week after completing two qFIT tests. The inclusion criteria were (1) asymptomatic people aged 45–75 years who were enrolled in the program for physical examination, (2) FIT positive with a cut-off value of 20 μg hemoglobin per gram stool (g), (3) participants who were willing to undergo colonoscopy. The exclusion criteria were (1) use of antibiotics within the past 3 months, (2) on a vegetarian diet, (3) had an invasive medical intervention within the past 3 months and (4) had a history of other types of cancer. Detailed records of all enrolled patients’ basic clinical information will be maintained, including age, gender, smoking history, pathological examination results, complete blood count data, and other relevant pathological findings (**Table 1**). The workflow was shown as follows (**Figure 1**).

**Table 1:** Clinical characteristics of the enrolled subjects.

Characteristic	Number	%
Gender		
Female	79 (79/197)	40.1%
Male	118 (118/197)	59.9%
Age		
<60	83 (83/197)	42.1%
≥60	114 (114/197)	57.9%
Patients		
Tumor	121 (121/197)	61.4%
Normal	76 (76/197)	38.6%
FIT		
<2	157 (157/197)	79.7%
≥2	40 (40/197)	20.3%
KRAS mutation		
Positive	63 (63/197)	32.0%
Negative	134 (134/197)	68.0%
M3 (△ct)		
<15.22	64 (64/197)	32.5%
15.22~20.03	54 (54/197)	27.4%
>20.03	79 (79/197)	40.1%
Methylation		
Positive	83 (83/197)	42.1%
Negative	114 (114/197)	57.9%

### 2.2 Fecal DNA extraction

After obtaining the fecal samples, a portion of the frozen samples is homogenized and centrifuged at 14,000 g for 5 minutes at room temperature using the NucleoSpin Soil Kit (Machery-Nagel GmbH & Co., Düren, Germany). The supernatant is used as the source for mt-sDNA. Genomic DNA was isolated and extracted from the fecal samples using the QIAamp DNA Stool Mini Kit according to the manufacturer’s instructions (Qiagen, Hilden, Germany). DNA concentration was measured with a NANO DROP 2000, and samples with concentrations below 10 ng/μl or OD260/OD280 ratios outside the range of 1.8–2.2 are re-extracted. All extracts were stored at −20°C until PCR amplification.

### 2.3 FIT test

FIT testing is performed using the Fujirebio quantitative fecal occult blood analyzer according to the manufacturer’s instructions. DNA from each fecal sample is sent in batches to separate laboratories for blinded analysis. The operators conducting the FIT tests have experience with at least 1,000 samples and remain blinded to other study-related results. Fecal samples with hemoglobin levels exceeding 100 ng/ml of buffer (or 20 μg hemoglobin per g of feces) are classified as positive. 100 ng/ml of buffer (or 20 μg hemoglobin per g of feces) were classified as positive. All enrolled samples undergo hemoglobin threshold classification, with thresholds set at 10 ng/ml, 50 ng/ml, 100 ng/ml, 200 ng/ml, and 400 ng/ml. 100 ng/ml was set as the final cutoff to determine a positive criterion.

### 2.4 Colonoscopy

The colonoscopy was performed by an experienced endoscopist, who examined the entire colon up to the cecum with an adequate withdrawal time. Both the pathologist and endoscopist were blinded to the qFIT results and study objectives. Adenomas were evaluated based on tumor number, size, and location: the right colon included the cecum, ascending colon, and proximal two-thirds of the transverse colon, while the left colon comprised the distal third of the transverse colon, descending colon, sigmoid colon, and rectum. Colonoscopy results with colitis, hyperplastic polyps, non-bleeding hemorrhoids, or diverticulosis were considered negative. Based on the colonoscopy findings, samples were classified as non-adenomatous polyps, other lesions, non-advanced adenomas, advanced adenomas, colorectal cancer, or advanced colorectal cancer.

### 2.5 KRAS mutation detection

Specifically, a KRAS mutation detection kit (PCR-capillary electrophoresis) was used to detect KRAS mutation using stool-based DNA, targeting common mutations such as Gly12Asp, Gly12Val, Gly12Ser, Gly12Cys, Gly12Ala, Gly12Arg, and Gly13Asp. The target gene was amplified via fluorescent quantitative PCR, and KRAS mutation status was determined using a capillary electrophoresis device (Yuewei). The PCR conditions were as follows: pre-denaturation at 95°C for 3 minutes (1 cycle), denaturation at 94°C for 15 seconds, followed by annealing and extension at 60°C for 45 seconds (45 cycles). The results were analyzed by the study’s biostatistician.

### 2.6 M3 detection

Fecal samples in patients were collected and stored in -20°C followed by DNA extraction. Then, specific primers targeting *M3* were designed and used for qPCR amplification. The primer sequences were as followings: Forward, 5’-AATGGGAATGGAGCGGATTC-3’; Reverse, 5’-CCTGCACCAGCTTATCGTCAA-3’. After normalization with the *ACTB* gene, M3 expression pattern was trisected equally into three parts based on the ΔCT value (high expression: <15.22, moderate expression: 15.22 - 20.03, and low expression: >20.03). Relative mRNA levels of the target genes were calculated using the 2^−ΔΔCT^ method.

### 2.7 Methylation detection

Stool-based DNA was used to detected methylation burden of *SDC2* gene using methylation-specific PCR. DNA extraction and bisulfite conversion were performed according to the manufacturer’s protocol. PCR was then used to detect methylation-specific fragments, with the internal control gene *ACTB* utilized to assess the adequacy of DNA quantity in the samples. Methylation-specific real-time PCR amplification was conducted on an ABI 7500 real-time PCR system (Thermo Fisher Scientific, MA, USA). The PCR protocol consisted of an initial denaturation at 94°C for 20 minutes, followed by 45 cycles at 62°C for 5 seconds, 55.5°C for 35 seconds, 93°C for 30 seconds, and a final extension at 40°C for 5 seconds. Positive and negative controls were included in each reaction to quantify the methylation burden. Methylation results were interpreted strictly in accordance with the manufacturer’s guidelines.

### 2.8 Statistical analysis

A receiver operating characteristic (ROC) curve analysis was conducted, and the area under the curve (AUC) was calculated to evaluate diagnostic performance. A 95% confidence interval for the AUC was estimated using a nonparametric approach. All statistical analyses were carried out using IBM SPSS Statistics version 23. P<0.05 was defined as the statistically significant differences.

## 3 Results

### 3.1 Diagnostic accuracy of single biomarkers

**Figure 2:**
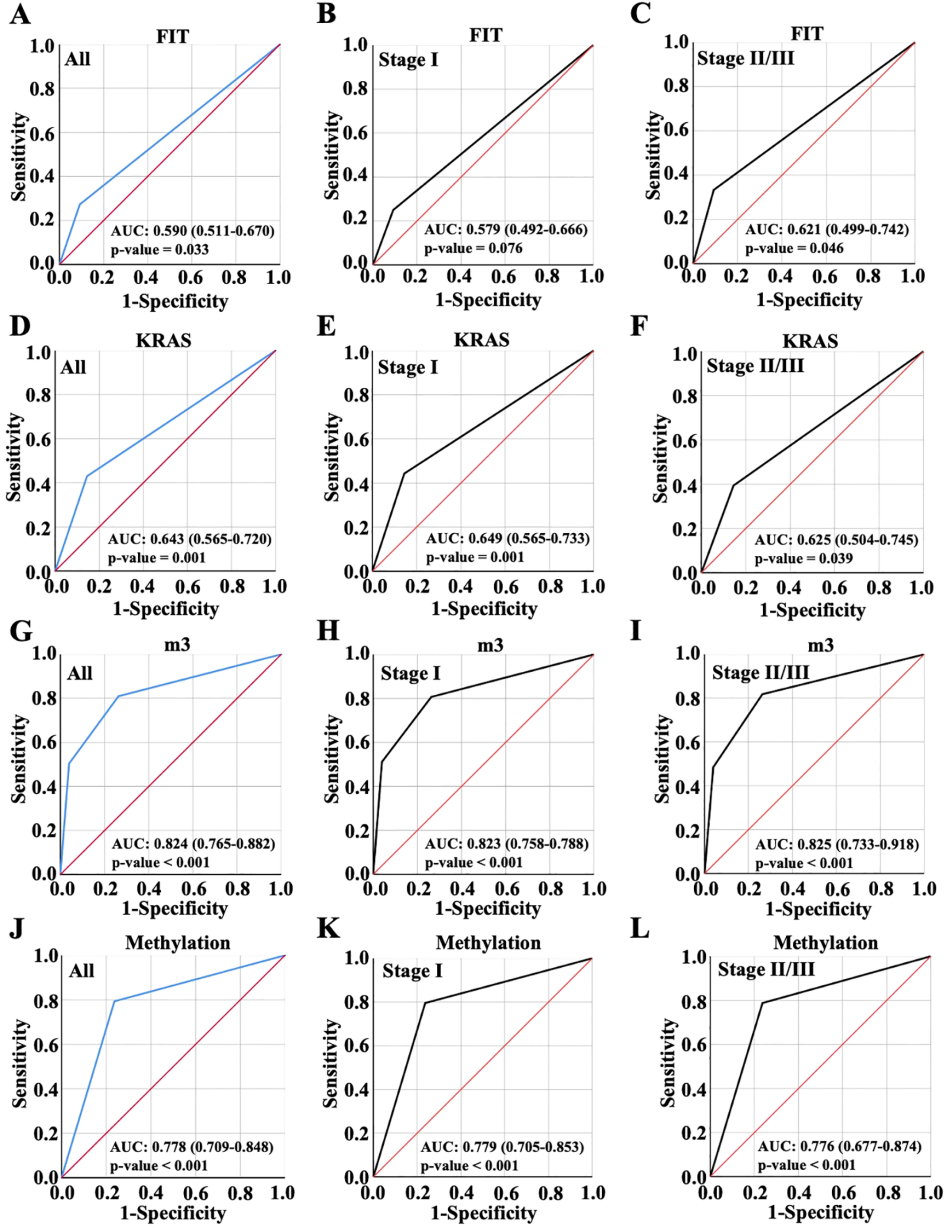
Diagnostic accuracy of single biomarkers. Diagnostic accuracy of FIT **(A-C)**, KRAS **(D-F)**, m3 **(G-I)** and Methylation **(J-L)** for all CRC patient diagnosis, and for early or advanced CRC diagnosis, which were displayed with ROC curve.

This study enrolled a total of 197 patients, consisting of 121 tumor cases and 76 normal cases. Four biomarkers were evaluated and analyzed: FIT, KRAS mutation, m3 and Methylation, the most recognized standard in clinical CRC diagnosis. In the results, there were 40 FIT positive patients, accounting for 20.3%. The diagnostic performance was demonstrated for FIT with AUC value 0.59 in all patients, 0.579 in patients with early CRC (Stage I, no significant) and 0.621 in patients with advanced CRC (Stage II/III) (**Figures 2A–C**). The incidence of KRAS mutation in CRC patients was also detected and 63 cases accounting for 32% were positive, including G12C^+^, G12V^+^ and G13D^+^ as the most common. AUC value with ROC curves for KRAS was 0.643 in all patients, 0.649 in patients with early CRC (Stage I) and 0.625 in patients with advanced CRC (Stage II/III) (**Figures 2D–F**). Moreover, m3 bacterial genes were also evaluated in all these patients. The results showed that high levels of m3 were observed in 64 cases with △ct values of amplified m3 gene lower than 15.22, 54 cases interposed between 15.22 and 20.03 and △ct values of m3 gene in 79 cases were above 20.3, indicating the lower expression of m3 gene. The diagnosis performance for m3 was 0.824 in all patients, 0.823 in patients with early CRC (Stage I) and 0.825 in patients with advanced CRC (Stage II/III) (**Figures 2G–I**). Furthermore, 83 cases were detected with DNA methylation, and the AUC value for methylation was 0.778 in all patients, 0.779 in patients with early CRC (Stage I) and 0.776 in patients with advanced CRC (Stage II/III) (**Figures 2J–L**). From evidence above, it was found that m3 showed the highest diagnosed performance among these four indicators while FIT exhibited the worst performance with AUC value lower than 0.6.

### 3.2 Diagnostic accuracy of random combinations of two variables

**Figure 3:**
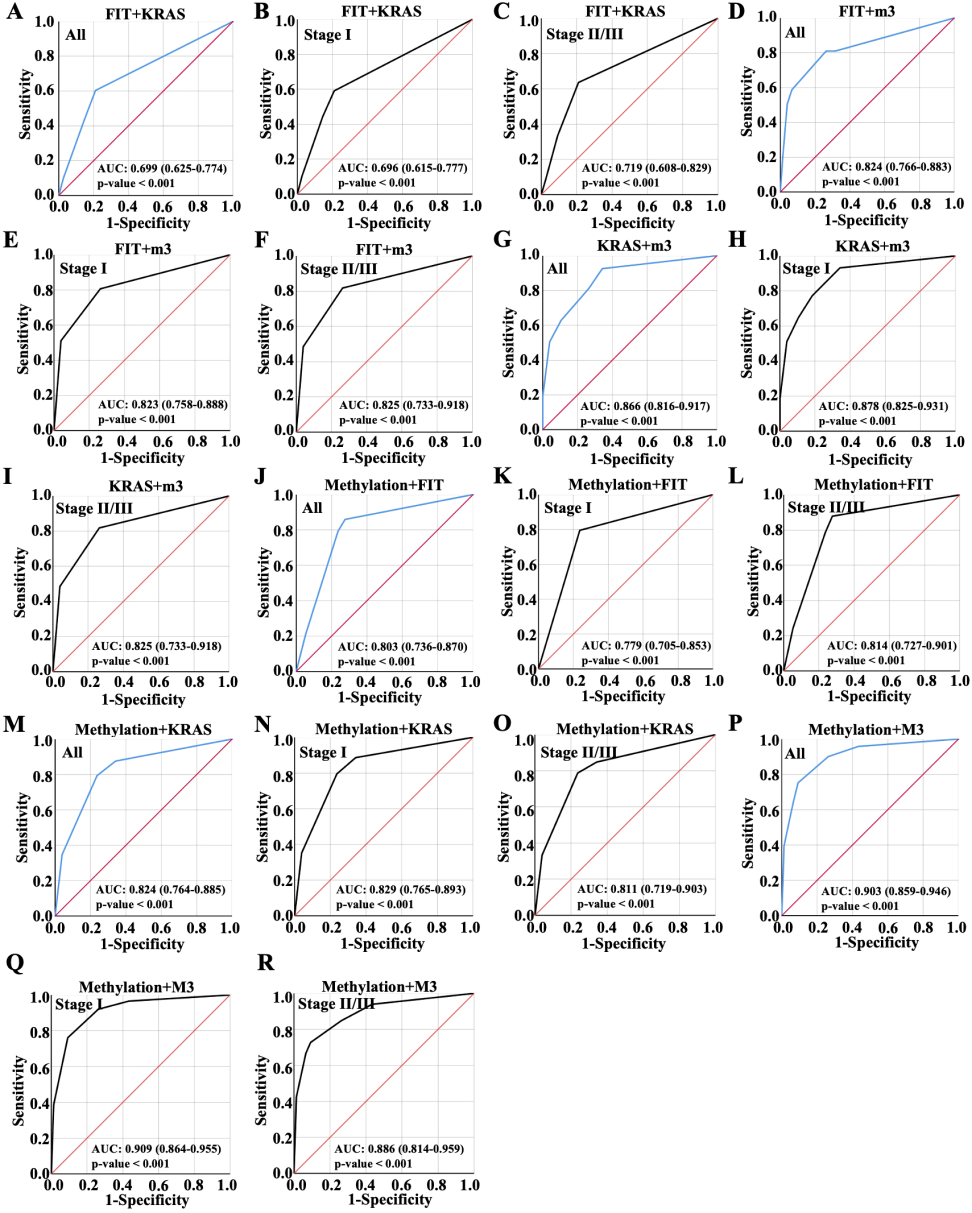
Diagnostic performance of random combinations of two variables, ranged by ROC. Diagnostic performance of the combination of **(A-C)** FIT and KRAS, **(D-F)** FIT and m3, **(G-I)** KRAS and m3, **(J-L)** Methylation and FIT, **(M-O)** Methylation and KRAS, **(P-R)** Methylation and m3 for all CRC patient diagnosis, and for early or advanced CRC diagnosis, which were displayed with ROC curve.

Next, the diagnosis performance of random combination of two variables were evaluated. As shown in **Figure 3**, it could be found that two indicators combination mostly showed better predictive performance than a single biomarker. The combination of FIT and KRAS demonstrated the lowest AUC value 0.699 in all patients, which was 0.696 in patients with early CRC and 0.719 in patients with advanced CRC (**Figures 3A–C**). Thus, the combination of FIT and KRAS were more suitable for patients with advanced CRC. Notably, the combination of FIT and m3 made no enhancement on the diagnosis performance with AUC value 0.824 equal to single m3 diagnosis (**Figure 3D** versus **Figure 2G**), and the diagnosis performance was 0.823 in patients with early CRC and 0.825 in patients with advanced CRC (**Figures 3E, F**). Once combined KRAS with m3, the AUC values elevated from 0.824 to 0.866 in all patients, which was 0.878 in patients with early CRC and 0.825 in patients with advanced CRC (**Figures 3G–I**), indicating that the combination of m3 and KRAS were more suitable for patients with early CRC. When combined methylation with FIT, KRAS and m3, respectively, the AUC values were 0.803, 0.824 and 0.903 in all patients (**Figures 3J, M, P**), respectively; For stage I CRC, the values were 0.779, 0.829 and 0.909 (**Figures 3K, N, Q**), which were 0.814, 0.811 and 0.886 in advanced CRC once combined methylation with FIT, KRAS and m3 (**Figures 3L, O, R**). Taken together, the combination of Methylation and m3 showed the highest AUC value for early CRC patients and advanced CRC patients, combinational marker for diagnosis showed better performance than single marker.

### 3.3 Diagnostic accuracy of random combinations of three variables

**Figure 4:**
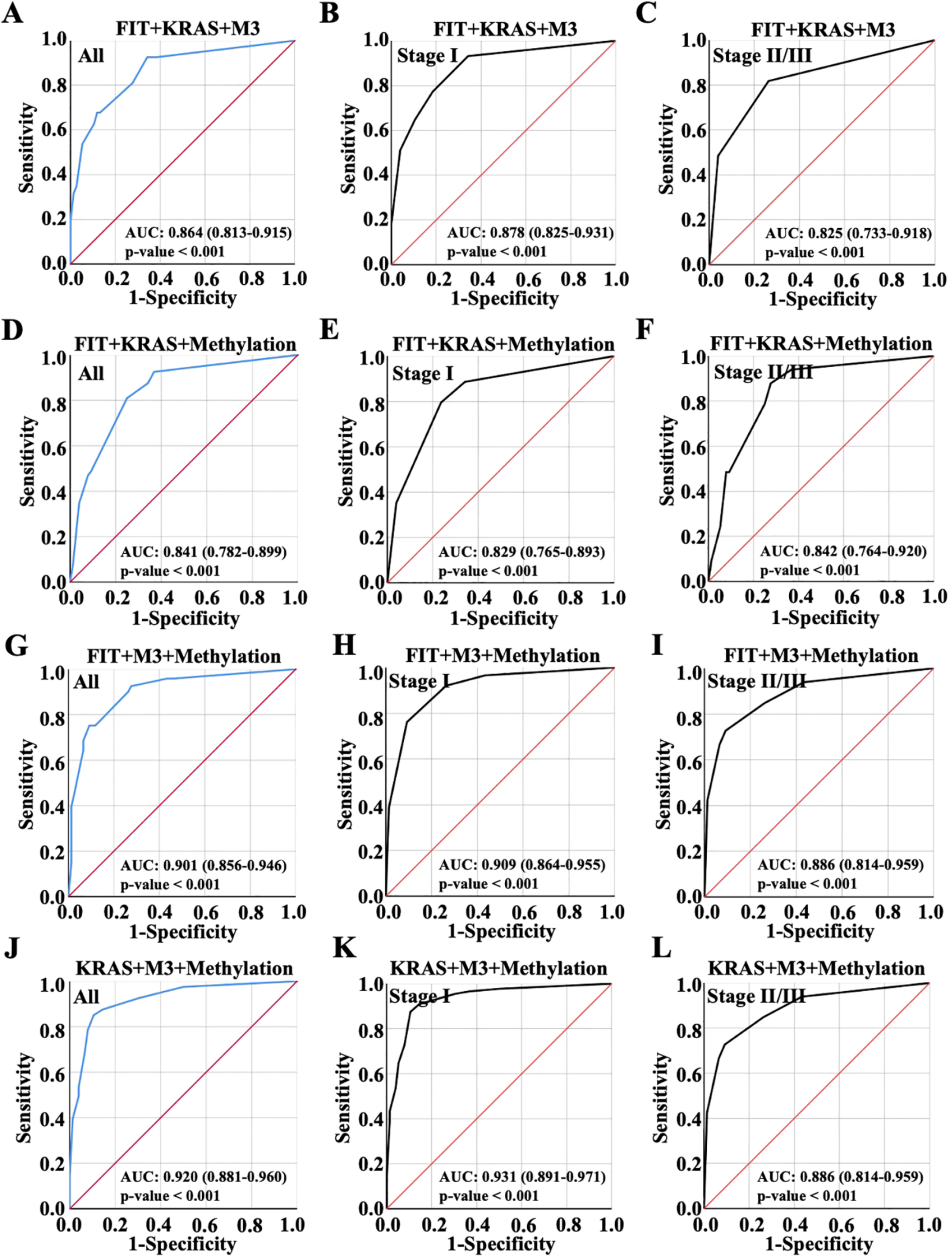
Diagnostic accuracy of random combinations of three variables presented by ROC curve. Diagnostic performance of the combination of **(A-C)** FIT, KRAS and m3 combination, **(D-F)** FIT, KRAS and Methylation combination, **(G-I)** FIT, m3 and Methylation combination, and **(J-L)** KRAS, m3 and Methylation combination for all CRC patient diagnosis, and for early or advanced CRC diagnosis, which were displayed with ROC curve.

Then, ROC analysis was performed with random integration of three variables. As observed, AUC value of the model of FIT, KRAS and M3 integration was 0.864 in all patients, exhibiting no improvement over the AUC value compared to KRAS and M3 combination; AUC value of the model of FIT, KRAS and M3 integration was 0.878 in early CRC and 0.825 in advanced CRC (**Figures 4A–C**). Besides, it was shown that FIT, KRAS and methylation combination showed slight enhancement on the diagnosis performance with AUC value 0.841 compared to the combination of any two variables between them, which was 0.829 in early CRC and 0.842 in advanced CRC (**Figures 4D–F**). Interestingly, the combination of FIT, M3 and Methylation combination showed a higher AUC value (0.901) than the groups of FIT plus M3 or FIT plus methylation, while lower than the combination of M3 plus methylation (**Figures 3**, **4G**); the combination of FIT, M3 and Methylation combination showed the AUC value 0.909 in early CRC and 0.886 in advanced CRC (**Figures 4H, I**). Noteworthy, KRAS, M3 and Methylation combination possessed the most predictive ability with AUC value elevated to 0.920 (Confidence Interval: 0.881-0.960), which were better than any pairwise combinations (**Figure 4J**); and this combination harbored the highest AUC value 0.931 in early CRC and 0.886 in advanced CRC (**Figures 4K, L**). It could be found from the results that the model of KRAS, M3 and methylation integration showed the most potential predictive ability for early CRC diagnosis.

### 3.4 Diagnostic accuracy of the overall combination of the four variables

**Figure 5:**
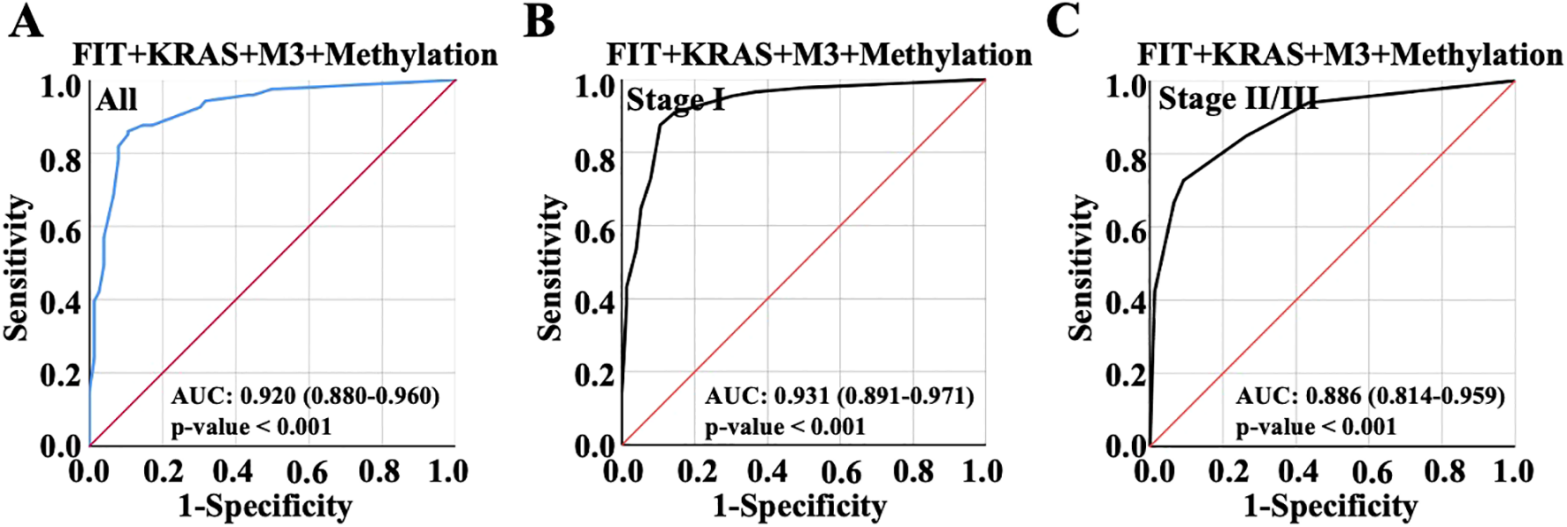
ROC curve was used to show the diagnostic performance with the overall combination of the four variables. **(A)** Diagnostic performance of the combination of FIT, KRAS, m3 and methylation for all CRC patient diagnosis. **(B)** Diagnostic performance of the combination of FIT, KRAS, m3 and methylation for early CRC patient diagnosis. **(C)** Diagnostic performance of the combination of FIT, KRAS, m3 and methylation for advanced CRC patient diagnosis.

Subsequently, the four indicators were combined to evaluate AUC value from the ROC curve, and the result showed AUC value was 0.920 (Confidence Interval: 0.880-0.960), making no evident alteration comparing to the model established with KRAS, M3 and Methylation combination, indicating that FIT might make no significant contribution to diagnosis performance when KRAS, M3 and Methylation were used to predict CRC (**Figure 5A**); The combination of four indicators showed the highest AUC value 0.931 in early CRC and 0.886 in advanced CRC (**Figures 5B, C**). Taking into account that a smaller AUC confidence interval indicated more credible of the AUC value, the combination of KRAS, M3 and Methylation presented a better predictive efficiency than the combination of four factors. In addition to that, the combination of three indicators was suitable for CRC diagnosis in view of the clinical timeliness.

## 4 Discussion

Screening, early diagnosis, and treatment have been validated as effective strategies for reducing the incidence and mortality of colorectal cancer (19). Colonoscopy combined with pathological examination remains the gold standard for colorectal cancer screening (20). However, due to its invasive nature, high cost, and the requirement for professional endoscopists, it is not feasible for large-scale population screening (21). Various diagnostic methods for colorectal cancer, such as FIT, are currently available, yet their effectiveness and specificity are limited (22). Multivariable approaches represent a promising strategy to enhance the performance of cancer risk assessment diagnostic tools and have received FDA approval (23). Multitarget stool DNA tests (Mt-sDNA) are an FDA approved, noninvasive, high-sensitivity CRC screening strategy (Cologuard). Although Mt-sDNA has documented superior sensitivity for CRC, high grade dysplasia, advanced adenoma, and sessile serrated adenoma/polyps compared to FIT alone, albeit with somewhat lower specificity (22, 24). This study focuses on colorectal cancer patients and employs multivariate analysis to elucidate the specificity and sensitivity of common diagnostic methods, including FIT, KRAS mutation, M3 and Methylation, thereby providing a reference for clinical screening and diagnosis of colorectal cancer.

FIT is one of the screening technologies recommended by international authoritative colorectal cancer screening guidelines (25). By detecting hidden blood in stool, it has been widely used in colorectal cancer screening programs worldwide (26). However, its sensitivity for small polyps is only 7.6%, even the latest stool DNA tests have a sensitivity of only 17.2% for small polyps (22). In our results, we found that the diagnostic performance of FIT was not as prominent compared to other indicators. A mutation in the KRAS gene, occurring early in cancer development, is recognized as a driver mutation (27). KRAS mutations are present in approximately 40-45% of colorectal cancer patients, with the most frequent mutations being G12V^+^, G12D^+^, G14D^+^, G12C^+^, and G12A^+^ (28). Advancements in point mutation detection technology have enhanced the analysis of biopsy specimens and enabled the evaluation of ctDNA in plasma and serum. These developments permit the early and precise detection of KRAS mutations in colorectal cancer patients (29). Due to the presence of KRAS mutations, this group of CRC patients requires more precise and personalized treatment (30). In our results, the diagnostic performance of KRAS mutation for colorectal cancer was also significant. M3 is the world’s first non-invasive colorectal cancer risk detection method capable of detecting both large and small polyps (17). It has a sensitivity and specificity of 94% and 85%, respectively, comparable to colonoscopy (31). In our results, M3 showed the best diagnostic performance in both early and advanced CRC patients compared to other indicators.

In clinical, combinations of two or more biomarkers are frequently used to enhance the accuracy and efficiency of disease diagnosis (32). Previous studies have reported that the combined use of blood markers outperforms single biomarkers for the clinical diagnosis of CRC (23). In our study, any two markers combination showed better diagnosis performances than single markers. Notably, our results indicated that the combination of all four markers did not significantly enhance diagnostic performance compared to the combination of KRAS, M3, and Methylation alone, suggesting that the combination of KRAS, M3, and Methylation might be the most effective strategy for CRC diagnosis, especially for early CRC patients.

However, this study also had some limitations. First, given that the study population was from a single region, the model lacked generalizability. Second, no validation model was constructed, and more clinical samples needed to be collected for biomarker validation. Thirdly, more clinical experiments needed to be included for verification of this combination. Fourthly, sample size and potential bias in patient selection should be further analyzed, and this findings in this study need for validation in prospective cohorts. Finally, some factors encountered during the practical implementation (including cost, scalability and turnaround time) should be considered and compared with the existing commercial tests (Cologuard combined with FIT and DNA methylation).

## 5 Conclusion

In summary, combination of four CRC diagnosis markers-FIT, KRAS, M3 and methylation showed enhanced diagnosis accuracy compared to univariate markers. KRAS, M3, and methylation integration exhibited the best diagnosis performance and have the potential to serve as decision-support tools in CRC diagnostic. Nevertheless, additional large-scale studies are required to validate the clinical utility of the developed diagnostic platform.

## Data Availability

The original contributions presented in the study are included in the article/supplementary material. Further inquiries can be directed to the corresponding author.

## References

[1] Marcellinaro R Spoletini D Grieco M Avella P Cappuccio M Troiano R. Colorectal cancer: current updates and future perspectives. J Clin Med. (2023) 13:1–12. doi: 10.3390/jcm13010040, PMID: 38202047 PMC10780254

[2] Brenner H Kloor M Pox CP. Colorectal cancer. Lancet. (2014) 383:1490–502. doi: 10.1016/S0140-6736(13)61649-9, PMID: 24225001

[3] Sung H Ferlay J Siegel RL Laversanne M Soerjomataram I Jemal A. Global cancer statistics 2020: GLOBOCAN estimates of incidence and mortality worldwide for 36 cancers in 185 countries. CA Cancer J Clin. (2021) 71:209–49. doi: 10.3322/caac.21660, PMID: 33538338

[4] Mo S Dai W Wang H Lan X Ma C Su Z. Early detection and prognosis prediction for colorectal cancer by circulating tumour DNA methylation haplotypes: A multicentre cohort study. EClinicalMedicine. (2023) 55:101717. doi: 10.1016/j.eclinm.2022.101717, PMID: 36386039 PMC9646872

[5] Chan SCH Liang JQ. Advances in tests for colorectal cancer screening and diagnosis. Expert Rev Mol Diagn. (2022) 22:449–60. doi: 10.1080/14737159.2022.2065197, PMID: 35400293

[6] Gallardo-Gomez M De Chiara L Alvarez-Chaver P Cubiella J. Colorectal cancer screening and diagnosis: omics-based technologies for development of a non-invasive blood-based method. Expert Rev Anticancer Ther. (2021) 21:723–38. doi: 10.1080/14737140.2021.1882858, PMID: 33507120

[7] Gupta S. Screening for colorectal cancer. Hematol Oncol Clin North Am. (2022) 36:393–414. doi: 10.1016/j.hoc.2022.02.001, PMID: 35501176 PMC9167799

[8] Vart G Banzi R Minozzi S. Comparing participation rates between immunochemical and guaiac faecal occult blood tests: a systematic review and meta-analysis. Prev Med. (2012) 55:87–92. doi: 10.1016/j.ypmed.2012.05.006, PMID: 22634386

[9] Vilkin A Rozen P Levi Z Waked A Maoz E Birkenfeld S. Performance characteristics and evaluation of an automated-developed and quantitative, immunochemical, fecal occult blood screening test. Am J Gastroenterol. (2005) 100:2519–25. doi: 10.1111/j.1572-0241.2005.00231.x, PMID: 16279909

[10] Goel A Boland CR. Epigenetics of colorectal cancer. Gastroenterology. (2012) 143:1442–60 e1. doi: 10.1053/j.gastro.2012.09.032, PMID: 23000599 PMC3611241

[11] Ness RM Llor X Abbass MA Bishu S Chen CT Cooper G. NCCN guidelines(R) insights: colorectal cancer screening, version 1.2024. J Natl Compr Canc Netw. (2024) 22:438–46. doi: 10.6004/jnccn.2024.0047, PMID: 39236750

[12] Benson AB Venook AP Adam M Chang G Chen YJ Ciombor KK. Colon cancer, version 3.2024, NCCN clinical practice guidelines in oncology. J Natl Compr Canc Netw. (2024) 22:1–26. doi: 10.6004/jnccn.2024.0029, PMID: 38862008

[13] Imamura Y Morikawa T Liao X Lochhead P Kuchiba A Yamauchi M. Specific mutations in KRAS codons 12 and 13, and patient prognosis in 1075 BRAF wild-type colorectal cancers. Clin Cancer Res. (2012) 18:4753–63. doi: 10.1158/1078-0432.CCR-11-3210, PMID: 22753589 PMC3624899

[14] Hinoue T Weisenberger DJ Lange CP Shen H Byun HM Van Den Berg D. Genome-scale analysis of aberrant DNA methylation in colorectal cancer. Genome Res. (2012) 22:271–82. doi: 10.1101/gr.117523.110, PMID: 21659424 PMC3266034

[15] Mitchell SM Ross JP Drew HR Ho T Brown GS Saunders NF. A panel of genes methylated with high frequency in colorectal cancer. BMC Cancer. (2014) 14:54. doi: 10.1186/1471-2407-14-54, PMID: 24485021 PMC3924905

[16] Chen H Li N Ren J Feng X Lyu Z Wei L. Participation and yield of a population-based colorectal cancer screening programme in China. Gut. (2019) 68:1450–7. doi: 10.1136/gutjnl-2018-317124, PMID: 30377193

[17] Liang JQ Li T Nakatsu G Chen YX Yau TO Chu E. A novel faecal Lachnoclostridium marker for the non-invasive diagnosis of colorectal adenoma and cancer. Gut. (2020) 69:1248–57. doi: 10.1136/gutjnl-2019-318532, PMID: 31776231 PMC7306980

[18] Miyata T Hayama T Ozawa T Nozawa K Misawa T Fukagawa T. Predicting prognosis in colorectal cancer patients with curative resection using albumin, lymphocyte count and RAS mutations. Sci Rep. (2024) 14:14428. doi: 10.1038/s41598-024-65457-8, PMID: 38910183 PMC11194255

[19] Maida M Macaluso FS Ianiro G Mangiola F Sinagra E Hold G. Screening of colorectal cancer: present and future. Expert Rev Anticancer Ther. (2017) 17:1131–46. doi: 10.1080/14737140.2017.1392243, PMID: 29022408

[20] Jayasinghe M Prathiraja O Caldera D Jena R Coffie-Pierre JA Silva MS. Colon cancer screening methods: 2023 update. Cureus. (2023) 15:e37509. doi: 10.7759/cureus.37509, PMID: 37193451 PMC10182334

[21] Bretthauer M Kaminski MF Loberg M Zauber AG Regula J Kuipers EJ. Population-based colonoscopy screening for colorectal cancer: A randomized clinical trial. JAMA Intern Med. (2016) 176:894–902. doi: 10.1001/jamainternmed.2016.0960, PMID: 27214731 PMC5333856

[22] Imperiale TF Ransohoff DF Itzkowitz SH Levin TR Lavin P Lidgard GP. Multitarget stool DNA testing for colorectal-cancer screening. N Engl J Med. (2014) 370:1287–97. doi: 10.1056/NEJMoa1311194, PMID: 24645800

[23] Voronova V Glybochko P Svistunov A Fomin V Kopylov P Tzarkov P. Diagnostic value of combinatorial markers in colorectal carcinoma. Front Oncol. (2020) 10:832. doi: 10.3389/fonc.2020.00832, PMID: 32528895 PMC7258084

[24] Redwood DG Asay ED Blake ID Sacco PE Christensen CM Sacco FD. Stool DNA testing for screening detection of colorectal neoplasia in alaska native people. Mayo Clin Proc. (2016) 91:61–70. doi: 10.1016/j.mayocp.2015.10.008, PMID: 26520415

[25] Chiu HM Chen SL Yen AM Chiu SY Fann JC Lee YC. Effectiveness of fecal immunochemical testing in reducing colorectal cancer mortality from the One Million Taiwanese Screening Program. Cancer. (2015) 121:3221–9. doi: 10.1002/cncr.29462, PMID: 25995082 PMC4676309

[26] Wisse PHA de Klaver W van Wifferen F van Maaren-Meijer FG van Ingen HE Meiqari L. The multitarget faecal immunochemical test for improving stool-based colorectal cancer screening programmes: a Dutch population-based, paired-design, intervention study. Lancet Oncol. (2024) 25:326–37. doi: 10.1016/S1470-2045(23)00651-4, PMID: 38346438

[27] Huang L Guo Z Wang F Fu L. KRAS mutation: from undruggable to druggable in cancer. Signal Transduct Target Ther. (2021) 6:386. doi: 10.1038/s41392-021-00780-4, PMID: 34776511 PMC8591115

[28] Hasbullah HH Sulong S Che Jalil NA Abdul Aziz AA Musa N Musa M. KRAS mutational profiles among colorectal cancer patients in the east coast of peninsular Malaysia. Diagn (Basel). (2023) 13:1–12. doi: 10.3390/diagnostics13050822, PMID: 36899966 PMC10001354

[29] Wen X Pu H Liu Q Guo Z Luo D. Circulating tumor DNA-A novel biomarker of tumor progression and its favorable detection techniques. Cancers (Basel). (2022) 14:1–36. doi: 10.3390/cancers14246025, PMID: 36551512 PMC9775401

[30] Zhu G Pei L Xia H Tang Q Bi F. Role of oncogenic KRAS in the prognosis, diagnosis and treatment of colorectal cancer. Mol Cancer. (2021) 20:143. doi: 10.1186/s12943-021-01441-4, PMID: 34742312 PMC8571891

[31] Liang JQ Zeng Y Kwok G Cheung CP Suen BY Ching JYL. Novel microbiome signatures for non-invasive diagnosis of adenoma recurrence after colonoscopic polypectomy. Aliment Pharmacol Ther. (2022) 55:847–55. doi: 10.1111/apt.16799, PMID: 35224756 PMC9303256

[32] Duffy MJ. Use of biomarkers in screening for cancer. Adv Exp Med Biol. (2015) 867:27–39. doi: 10.1007/978-94-017-7215-0_3, PMID: 26530358

